# Modulatory Effect of Acupuncture at Waiguan (TE5) on the Functional Connectivity of the Central Nervous System of Patients with Ischemic Stroke in the Left Basal Ganglia

**DOI:** 10.1371/journal.pone.0096777

**Published:** 2014-06-13

**Authors:** Junqi Chen, Jizhou Wang, Yong Huang, Xinsheng Lai, Chunzhi Tang, Junjun Yang, Junxian Wu, Tongjun Zeng, Shanshan Qu

**Affiliations:** 1 Department of Rehabilitation, The Third Affiliated Hospital of Southern Medical University, Guangzhou, China; 2 The First Clinical Medical School, Southern Medical University, Guangzhou, China; 3 School of Traditional Chinese Medicine, Southern Medical University, Guangzhou, China; 4 School of Acupuncture and Rehabilitation, Guangzhou University of Traditional Chinese Medicine, Guangzhou, China; 5 Department of Acupuncture and Moxibustion, Shantou Central Hospital, Shantou, China; 6 The First People’s Hospital of Shunde, Foshan, China; VU University Medical Center, Netherlands

## Abstract

**Objective:**

To study the influence of acupuncture at Waiguan (TE5) on the functional connectivity of the central nervous system of patients with ischemic stroke.

**Methods:**

Twenty-four patients with ischemic stroke in the left basal ganglia were randomized based on gender to receive TE5 acupuncture (n = 12) or nonacupoint acupuncture (n = 12). Each group underwent sham acupuncture and then verum acupuncture while being scanned with functional magnetic resonance imaging. Six regions of interest (ROI) were defined, including bilateral motor, somatosensory, and bilateral basal ganglia areas. The functional connectivity between these ROIs and all voxels of the brain was analyzed in Analysis of Functional NeuroImages(AFNI) to explore the differences between verum acupuncture and sham acupuncture at TE5 and between TE5 acupuncture and nonacupoint acupuncture. The participants were blinded to the allocation.

**Result:**

The effect of acupuncture on six seed-associated networks was explored. The result demonstrated that acupuncture at Waiguan (TE5) can regulate the sensorimotor network of the ipsilesional hemisphere, stimulate the contralesional sensorimotor network, increase cooperation of bilateral sensorimotor networks, and change the synchronization between the cerebellum and cerebrum. Furthermore, a lot of differences of effect existed between verum acupuncture and sham acupuncture at TE5, but there was little difference between TE5 acupuncture and nonacupoint acupuncture.

**Conclusion:**

The modulation of synchronizations between different regions within different brain networks might be the mechanism of acupuncture at Waiguan (TE5). Stimulation of the contralesional sensorimotor network and increase of cooperation of bilateral hemispheres imply a compensatory effect of the intact hemisphere, whereas changes in synchronization might influence the sensorimotor function of the affected side of the body.

**Trial Registration:**

Chinese Clinical Trial Registry ChiCTR-ONRC-08000255

## Introduction

Acupuncture is one of the most widely used alternative treatments whose curative effect has been recognized and approved by the World Health Organization [1]. In traditional Chinese medicine, it is believed that acupuncture can affect the energy flow through the body which in turn modulates functions of the whole body system. However, the mechanism of its effect has not been well characterized to date. Clinical and experimental studies suggest that the modulatory effect of acupuncture might be mediated via the central and peripheral nervous systems [2], [3].

Functional magnetic resonance imaging (fMRI) is an efficient, noninvasive method of studying the mechanism by which acupuncture affects the central nervous system (CNS). Many fMRI studies indicated that acupuncture can activate or deactivate certain areas of the brain related to a corresponding disease or function [4]–[12]. Meanwhile, its effect seems to be relevant to the regulation of brain networks, such as the default mode network, sensorimotor network, amygdala-associated network, and vision network [6], [13]–[15]. Distinct from the conventional fMRI, functional connectivity MRI (fcMRI) can be used to detect the temporal correlation of the blood oxygen level–dependent (BOLD) signals of spatially remote brain regions. The connectivity of two areas, including functional connectivity and effective connectivity, depicts the cooperation pattern of these areas. Functional connectivity is the pattern of statistical dependency resulting from the nonlinear dynamics of neurons and neuronal populations within the neuroanatomical substrate [16], which reflects the existence and the strength of the connectivity between remote brain regions.

Patients with stroke in the basal ganglia have significant deficiency in the sensorimotor function of the contralesional side of their body. To date, researchers have found several relevant changes in the CNS of these patients, such as changes in the effective connectivity of core motor areas [17]. Recovery seems to be related to the extent of connectivity between the ipsilesional primary motor area and contralesional postcentral gyrus, change in the topological structure, centrality of the ipsilesional primary sensorimotor area, recruitment of bilateral somatosensory association areas and contralesional SII, and activation of the contralesional cerebellum [18], [19]. Similarly, the abnormal function of many regions of the brain is also crucial in the deficiency in somatosensory function [20]–[22]. The sensorimotor function is regulated by complex functional networks formed by the neuronal populations of the cortex and subcortex [23]. Thus, ischemic stroke lesions may affect the functional network architecture in both hemispheres [24]–[28] and break the balance of the network, which in turn causes the deficiency in sensorimotor function.

Acupuncture is one of the most important treatments for stroke rehabilitation. Several studies demonstrated that acupuncture not only regulates the functional state and connectivity of sensorimotor areas of normal people [9], [14], [29] but also affects the functional state of the bilateral sensorimotor cortex of stroke patients [30], [31].

TE5 is an important traditional acupuncture point common in the treatment of stroke-related motor, neurological and autonomic nerve problems in clinical practice [32]. We hypothesize that acupuncture at TE5 has a specific influence on the functional networks, including sensorimotor areas, of the CNS to improve the sensorimotor function of the body [33].

Twenty-four patients with ischemic stroke in the left basal ganglia were recruited to investigate this hypothesis. Data extracted from fMRI were assessed with seed-based analysis to discover the differences between TE5 verum acupuncture and nonacupoint acupuncture and between TE5 verum acupuncture and TE5 sham acupuncture. Results of this study elucidate the specific influence of acupuncture at TE5 on sensorimotor networks.

## Methods

### Subject

The study was carried out during October 2008 and August 2010 in the Imaging Center of Nanfang Hospital, Guangzhou, China. Twenty-four patients admitted to the First Affiliated Hospital of Guangzhou University of Chinese Medicine and matching the diagnostic criteria of ischemic stroke in ICD-9 434 and ICD-8 433 [34] were screened based on the following inclusion criteria: ischemic stroke in the left basal ganglia that occurred more than a month ago but less than a year, significant right hemiplegia (the score of the muscle strength of the upper limb and the lower limb ≤4), stable condition and receiving usual treatment (including antiplatelet medicine like aspirin and clopidogrel, and lipid-lowering drugs like statins et al.), right handedness, naive to acupuncture or not being treated by acupuncture for at least 4 weeks, no severe aphasia, no previous neurological or psychiatric disease, and no coagulation or other severe diseases. The experimental protocol was approved by the Ethical Committee of the First Affiliated Hospital of Guangzhou University of Chinese Medicine. This study was registered on the Chinese Clinical Trial Registry (http://www.chictr.org, ChiCTR-ONRC-08000255). All patients signed a written informed consent.

The participants were randomly (using random number table) divided into two groups of 12: Waiguan (TE5) group and nonacupoint group. Each group underwent sham acupuncture and then verum acupuncture.

### Experiment Design

**Figure 1:**
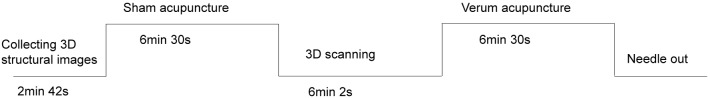
Stimulation and scanning pattern.

The fMRI brain scan was conducted on a 3.0-T whole-body scanner (GE Signa) with a standard head coil. The participants were prevented from experiencing auditory and visual activities via earplugs and eyeshades, respectively. The scanning procedure (Fig. 1) began after the participants rested on the bed for 5 min. The participants in each group were given sham acupuncture stimulus and then verum acupuncture while being simultaneously scanned. Each stimulus lasted for 6 min and 30 s. The two stimuli had an interval lasting for 6 min and 2 s, which was considered sufficient for restoring the sensitivity of cutaneous sensory receptor. The participants were blinded as to which stimulus was given to them; they were only aware of receiving acupuncture. The experiment operator, data analyst, and researcher were strictly separated from each other.

### Verum Acupuncture Stimulation

**Figure 2:**
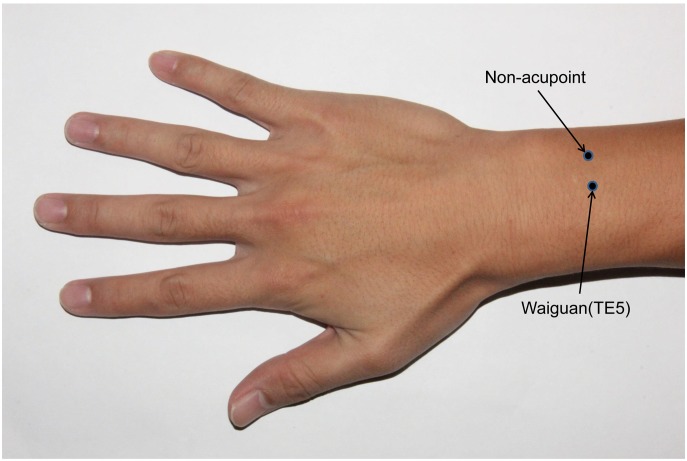
*Waiguan* and nonacupoint on the right forearm.

TE5 is located on the dorsal aspect of the forearm at midpoint of the interosseous space between the radius and the ulna, 2 cun proximal to the dorsal wrist crease, whereas the nonacupoint is medial to TE5 at midpoint between the two meridians, i.e. triple energizer meridian and small intestine meridian (Fig. 2). During stimulation, a sterile silver needle 0.30 mm in diameter and 40 mm in length (tube purchased from Dongbang AcuPrime Co. and needle from Zhongyan Taihe Co., Beijing, China) was inserted vertically into the skin at a depth of 15±2 mm. The needle was twisted for ±180° evenly at a frequency of 60 circles per min after the needling sensation (de qi), i.e., the feeling of increased resistance to further insertion, was assured by the acupuncturist.

### Sham Acupuncture Stimulation

In this study, sham acupuncture served as the tactile control. Tactile stimulation has been widely used as a noninvasive control for acupuncture neuroimaging studies. The procedure involved pushing the end of the needle with its tip out of the tube within 1 mm and touching the skin.

Acupuncture was conducted manually by an experienced acupuncturist. During experimental stimulation, the subjects were required to keep quiet and remain calm without speaking.

### fMRI Scan

After the patients rested on the bed for 5 min, 3D anatomy images were collected with a T1-weighted 3D gradient echo-pulse fast spin sequence, with axial view T1 fluid-attenuated-inversion-recovery scan. The exact scanning parameters were as follows: TR = 2.3 s, TE = 21 ms, TI = 920 ms, slice thickness = 6.0 mm, gap = 1.0 mm, 20 layers for a total of 2 min and 45 s, field of view (FOV) = 240×180 mm^2^, matrix = 320×256, number of excitations (NEX) = 2, echo train length = 9, and band width = 50. During the acupuncture stimulation, BOLD functional images were collected with a T2-weighted single-shot, gradient-recalled echo-planar imaging sequence. The exact scanning parameters were as follows: TR = 3 s, TE = 20 ms, flip angle = 90°, FOV = 240×240 mm^2^, slice thickness = 6.0 mm, slice gap = 1.0 mm, matrix = 96×96, NEX = 1, phase per location = 130, and 2600 phases per 6 min and 30s.

### Data Analysis

The preprocessing steps were implemented in AFNI (Cox, 1996; http://afni.nimh.nih.gov/afni). The functional images from each run were aligned, slice timing corrected, temporally standardized, space smoothed (6 mm full width at half maximum Gaussian kernel), and transformed into Talairach space (Talairach and Tournoux, 1988).

For each subject, we analyzed the functional connectivity using the average activity of a seed region defined by the anatomical template in AFNI to find other voxels in the brain that behaved similarly. The ROIs (seeds) selected were areas related to motor ability [Brodmann area (BA) 4 and BA6], sensations (BA1, BA2, BA3, BA5, and BA7), and basal ganglia area. The objective was to find the changes in correlations between these regions and other parts of the brain, which might affect the function of motor ability and sensations. The low-frequency BOLD correlations (0.01 Hz to 0.1 Hz) or functional connectivity between the time series of a given seed and that of all voxels in the brain was partially correlated by Pearson correlation analysis with covariance of head motion and time series from white matter and cerebrospinal fluid. Individual correlation coefficient maps for both the acupuncture and tactile conditions were generated and transformed to Fisher’s z-distribution for group-level analysis. All the resultant t-maps were set to the threshold level of P<0.05. Multiple-comparison error was corrected with Monte Carlo simulation.

## Results

### Baseline Data

**Figure 3:**
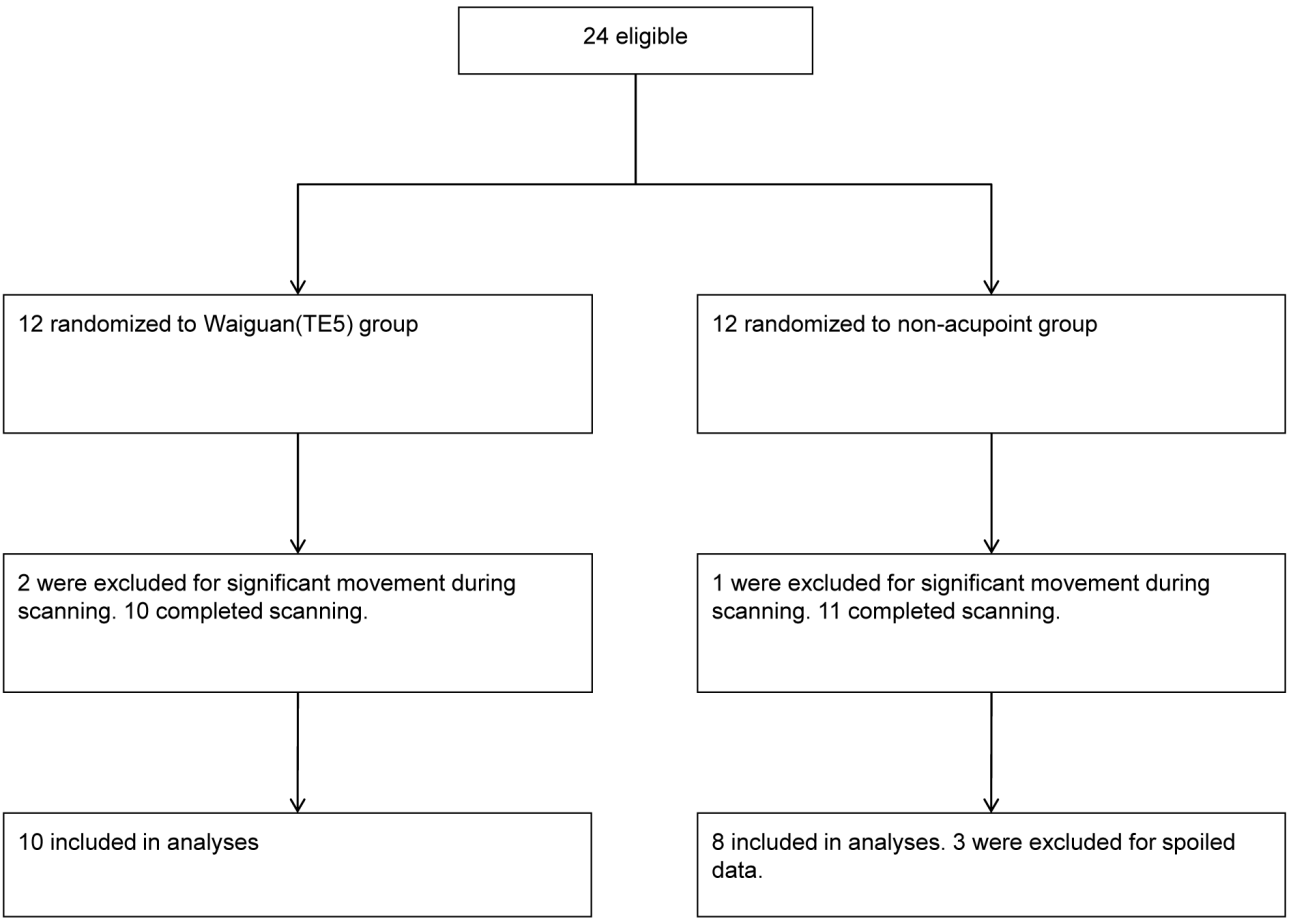
Consort flow diagram.

Two participants from the Waiguan (TE5) group and one participant from the nonacupoint group were excluded for significant movement during the scan, and three participants from the nonacupoint group were excluded for spoiled data (Fig. 3). No significant difference was found in the baseline data of the remaining 18 participants (Table 1).

**Table 1:** Baseline data.

Items	Waiguan (TE5) (n = 10)	Nonacupoint (n = 8)	Statistics	P
Gender (M/F, n)	9/1	7/1		1*
Age	56.10±5.53	58.50±7.05	t = −0.811	0.429
Duration, months	5.30±3.71	3.38±3.29	t = 1.148	0.268
CSS score	18.20±4.02	17.13±4.76	t = 0.520	0.611
Hypertension (Yes/No, n)	9/1	6/2		0.559*
Diabetes mellitus (Yes/No, n)	2/8	1/7		1*

The P values with “*”were obtained using Fisher’s Exact Test, whereas the rest were the result of independent samples t-test.

### Functional Connectivity

**Figure 4:**
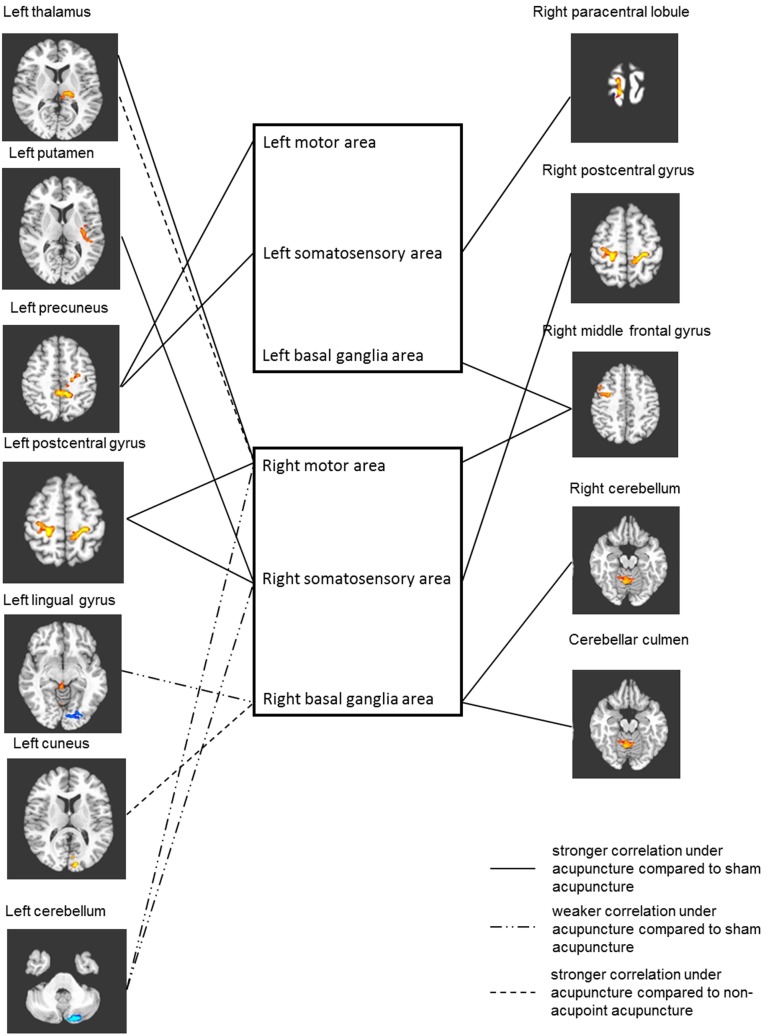
Differences of seed associated networks between ROIs from the left hemisphere and the right hemisphere. Full line represents stronger correlation under acupuncture compared with sham acupuncture, whereas the “dash–dot–dot” line represents weaker correlation. Dash line stands for weaker correlation compared with nonacupoint acupuncture (P<0.05, multiple comparison error corrected using Monte Carlo simulation). Regions of the left hemisphere and right hemisphere that had significant differences in correlation with seeds are placed on left side and right side, respectively, and ROIs in the same box are from the same hemisphere.

The different effects of TE5 verum acupuncture, TE5 sham acupuncture, and nonacupoint acupuncture on six seed-associated networks were observed through seed-based analysis (Table 2 and Fig. 4).

**Table 2:** Localization of the acupuncture specific effects by comparing TE5 verum acupuncture (Group A) vs. TE5 sham acupuncture (Group B) and TE5 verum acupuncture (Group A) vs. nonacupoint acupuncture (Group C).

ROI	Group A vs. Group B							Group A vs. Group C						
	Anatomicalstructures	BA	X	Y	Z	vox	t	Anatomicalstructures	BA	X	Y	Z	vox	t
Left motor area	Left precuneus	7	−10	−40	47	69	3.5							
Leftsomatosensoryarea	Left precuneus	5,7	−1	−37	47	48	5.2							
	Right paracentrallobule	4*	23	−37	50	33	3.5							
Left basalganglia area	Right middlefrontal gyrus	6	32	5	50	53	4.2							
Right motorarea	Left postcentralgyrus	3	−25	−31	50	133	4.2	Left thalamus	N/A	−16	−22	5	31	3.8
	Left thalamus	N/A	−16	−25	8	66	4							
	Right middle frontal gyrus	6	32	−7	47	40	5.8							
	Left cerebellar	N/A	−19	−79	−34	38	−4.4							
Rightsomatosensoryarea	Right postcentralgyrus	3	20	−31	50	121	4.4							
	Left postcentral gyrus	3	−28	−31	50	69	3.5							
	Left putamen	N/A	−31	−16	8	50	4							
	Left cerebellar	N/A	−19	−79	−34	44	−7.2							
Right basalganglia area	Right cerebellar	N/A	5	−55	−25	113	4.9	Left cuneus	18	−10	−85	17	41	5.2
	Cerebellarculmen	N/A	2	−34	−1	47	5.2							
	Left lingualgyrus	18	−25	−79	−4	44	−3.1							

Abbreviation: BA, Brodmann area; Vox, voxel (represents the number of voxels); N/A, not available (means that the peak voxel was out of the BA zone). The BA area marked by “*”was corrected by a neurological physician.

The correlation between the left precuneus and the left motor area was stronger under acupuncture than under the tactile control. Greater correlation was found between the left precuneus and the left somatosensory area, the latter being the seed. Stronger correlation was found between the left basal ganglia area and the right middle frontal gyrus, with the former being the seed.

The three seed-associated networks of the ROIs in the right hemisphere were more complicated than that in the left hemisphere. The correlation between the right motor area and the left postcentral gyrus, right middle frontal gyrus, and left thalamus was stronger under acupuncture than under the tactile control. Meanwhile, the correlation between the right somatosensory area and the bilateral postcentral gyrus and left putamen was stronger under acupuncture than under the tactile control. The right cerebellum and cerebellar culmen had greater connectivity with the right basal ganglia, whereas the left cerebellum had weaker correlation with the right motor and somatosensory areas.

Unlike the many differences demonstrated in the comparison of TE5 verum acupuncture and TE5 sham acupuncture stated before, only the left thalamus and left cuneus showed greater connectivity with the right motor area and right basal ganglia, respectively, when TE5 verum acupuncture was compared with nonacupoint acupuncture. Interestingly, stronger connectivity between left thalamus and right motor area was also aroused by verum acupuncture relative to sham acupuncture.

## Discussion

### Specific Effects of TE5 Acupuncture Compared with TE5 Sham Acupuncture

First, the left precuneus showed stronger connectivity with both the left motor area and left somatosensory area. Anatomical connectivity exists between the precuneus and the sensorimotor cortex and subcortex [35]. A clinical study indicated that acupuncture can activate regions of ipsilesional hemisphere, which is correlated with the extent of rehabilitation [31]. Taking the anatomical connectivity and this clinical study into account, we hypothesize that acupuncture at TE5 can change the functional connectivity among ipsilesional neuronal populations of sensorimotor cortexes and subcortexes, leading to the modulation of the sensorimotor network, which might have a positive effect on the recovery of the affected side of the body. In addition, the precuneus was also found to have strong correlation with other brain regions [11], implying that the precuneus affects the efficiency of acupuncture.

Second, the correlation between the right sensorimotor areas and the left somatosensory area (left postcentral gyrus), right somatosensory area (right postcentral gyrus), right motor area [right middle frontal gyrus (BA6, premotor area)], and left basal ganglia area (left thalamus, left putamen) was stronger under acupuncture than under sham acupuncture. Meanwhile, a clinical study suggested that the regions activated or deactivated by acupuncture at TE5 of the affected forearm are mainly the components of the contralesional hemisphere [30]. Hence, the modulatory effects of acupuncture at TE5 might be attributed to the enhancement of compensatory process by the redistribution of functions to the sensorimotor areas of the contralesional hemisphere and to the increase in the cooperation of functioning between the bilateral sensorimotor areas. Our results were similar to those of Johansen-Berg [36], [37] and Grefkes [17], who indicated that the sensorimotor areas of the contralesional hemisphere might have a role in modulating the function of the affected side of the body. Moreover, Grefkes et al. also found that the contralesional M1 of patients with stroke in the left basal ganglia was not only significantly activated while moving the affected limb but also exerted a negative influence on the ipsilesional M1. This result was not observed in healthy people. According to these studies, the negative influence can be attributed to the abnormal coupling between bilateral M1 caused by the damage of basal ganglia. Our analysis methods are different from theirs. Hence, ensuring whether or not the change in connectivity between the bilateral sensorimotor areas implies a modulatory effect on the coupling of these areas warrants further study.

Moreover, the connectivity between the left cerebellar cortex and the right motor area was weaker. The left cerebellum and the right motor area cooperate to control the left limb. Therefore, the result might reflect a decrease in the modulation of the sensorimotor function of the left limb. Meanwhile, the cerebella medial zone and the right cerebellar intermediate zone, which regulate the tension and cooperation of the body and proximal limb muscles and modulate the motor function of the right distal limb muscle, respectively, were more correlated with the right basal ganglia under acupuncture than under the tactile control. This result implies that the cooperation possibly aroused by the acupuncture between the right basal ganglia area and the right cerebellum might have a modulatory effect on the sensorimotor function of the right side (affected side) of the body. Moreover, the correlation between the right basal ganglia and the cerebellar culmen might regulate the sensorimotor function of both sides. Similar to our study, some clinical studies found that the functional recovery of patients improves with more activity in the contralesional cerebellum and with weaker centrality of the ipsilesional cerebellum [18], [38]. Nevertheless, the reasons behind why the correlations between the cerebellum and other sensorimotor areas did not show significant differences need further study. In general, acupuncture at the right TE5 of stroke patients attenuates the cooperation of the right hemisphere and the left cerebellum responsible in modulating the sensorimotor function of the unaffected limb while enhancing the cooperation of the right hemisphere and the right cerebellar to promote the regulation of the sensorimotor function of the right side (affected side) body.

Finally, a difference in correlation was also found between the visual cortex and the ROI, apart from the one between the sensorimotor function-associated areas and ROIs. Lingual gyrus is a part of the visual cortex, which also has functions in word comprehension and in the regulation of emotion and motor ability. Hence, the weaker correlation between these areas implies an attenuated cooperation on these functions with the use of acupuncture. However, a clinical study reported that acupuncture at TE5 can activate the bilateral occipital lobe compared with the baseline; however, the activation is not significant relative to that of tactile stimulation [30]. One reason that could account for this result is the different stimulation patterns of the verum and sham acupuncture on the visual cortex, such that distinction was found in its connectivity with the right basal ganglia, but not in activity. However, further studies are needed to investigate the exact benefit of this difference for the rehabilitation of stroke patients.

### Specific Effects of TE5 Acupuncture Compared with Nonacupoint Acupuncture

Only the left thalamus and the left cuneus exhibited greater connectivity with the right motor area and the right basal ganglia, respectively, when TE5 acupuncture was compared with nonacupoint acupuncture. The stronger correlation between the left thalamus and the right motor areas implies an enhancement in the compensatory effect of the right intact sensorimotor areas. Interestingly, stronger connectivity between the left thalamus and the right motor area was also observed when TE5 acupuncture was compared with sham acupuncture. Thus, it can be a relatively specific effect of acupuncture at TE5. As stated above, the difference in the correlation between the left cuneus and the right basal ganglia might have resulted from the distinct stimulation pattern on the visual cortex and the augmented correlation under acupuncture when compared with nonacupoint acupuncture. When referring to the comparison between acupuncture and sham acupuncture, the correlation between the left visual cortex and the right basal ganglia was attenuated by acupuncture. This seemingly conflict needs to be clarified in further study with more exact data.

We propose the fact that little difference was found between the two groups might be related to the location of the nonacupoint. Locating the acupoint and the nonacupoint is rather arbitrary, and the surface area and spatial structure of an acupoint vary with the acupoint [39]–[41]. Thus, stimulating the nonacupoint and TE5 might have similar effects since these two points were close to each other, and the small difference in location might contribute to the differences in functional connectivity. Another possibility is that the nonacupoint is a new acupoint that might have a similar effect because it is situated close to TE5. Moreover, the fact that little difference was found might proceed from the analysis of acupuncture’s instant effects. Acupuncture’s long lasting effects have been demonstrated by many researchers. Hence more difference might be found if these effects were measured [42]. Although nonacupoint is commonly used as a control, investigating how to design a nonacupoint to reflect the actual effect of acupuncture is necessary to avoid the influence of nervous tissue, connective tissue, and other anatomic structures.

### Characteristics of Connectivity Difference

Differences in connectivity were observed in several pairs of brain regions when TE5 acupuncture was compared with TE5 sham acupuncture and nonacupoint acupuncture (Table 2 and Fig. 4). A pair of brain regions consisted of an ROI and another brain region. Most of the ROIs within these pairs were from the right hemisphere, whereas the other parts of the pairs were mainly areas of the left hemisphere and the left cerebellum. This type of distribution pattern implies that the main effect of acupuncture at TE5 of stroke patients might be the enhancement of the association between the two hemispheres, which cooperate to modulate the sensorimotor function of the affected side. Moreover, it could also be a sign of increasing the compensatory effect of the unaffected side of the brain.

### Limitations

The sample size of our study was relatively small, and all participants were diagnosed of ischemic stroke in the left basal ganglia. We were unable to distinguish the exact regions of stroke. Therefore, our results only gave a preview on the mechanism of the effect of acupuncture on the CNS of patients with left basal ganglia ischemic stroke. In addition, the functional connectivity only refers to the correlation of the BOLD signal of two distinct brain regions, which indirectly reflects the correlation or cooperation of the neuronal activities. However, the correlation cannot indicate the effect passage of the neural function. Hence, further studies on the topic should use effective connectivity or graph theory, which is effective in revealing the direction of neural function, to enhance our understanding of the effect of acupuncture at TE5. We only focused on the connectivity between ROIs and all the voxels of the brain. Thus, the connectivity between the voxels that showed significant change in correlation with ROIs and the connectivity between regions other than the chosen ROIs and all voxels of the whole brain were not studied. Acupuncture is known to have a long lasting effect [13], [15], [43], and this characteristic was not investigated in this study. Further studies are warranted to explore the sustained effects of acupuncture on functional connectivity and its correlation with function recovery.

The commonly used TE5 sham acupuncture and nonacupoint acupuncture served as controls. However, previous research indicated that sham acupuncture and nonacupoint acupuncture have certain curative effects [44], [45]. Therefore, the present study also neglected the connectivity, which did not show significant difference between groups that might influence the effect of acupuncture. A more suitable control needs to be adopted to reflect the exact effects of acupuncture.

## Conclusion

The present study found that acupuncture helps regulate the functional connectivity between the sensorimotor areas of intra-hemisphere and inter-hemispheres as well as between the cerebellum and cerebrum. Results indicate that the compensatory effect of the intact sensorimotor network of contralesional hemisphere might be enhanced. The cooperation of the sensorimotor network of the ipsilesional hemisphere might be augmented. The impact on the functional connectivity between the cerebellum and cerebrum might also be important for the acupuncture’s effects. The modulation of synchronizations between different regions within different brain networks might be the mechanism of acupuncture at Waiguan (TE5). A number of limitations were discussed.

## Supporting Information

Checklist S1 Stricta checklist.
(DOCX)

Protocol S1 Trial protocol.
(DOC)

## References

[1] NIH Consensus Conference. Acupuncture. JAMA 280: 1518–1524. 9809733

[2] Cheng XN (2000) Chinese Acupuncture and Moxibustion. Beijing: People’s Medical Publishing House.

[3] Han JS (2003) Acupuncture: neuropeptide release produced by electrical stimulation of different frequencies. Trends Neurosci 26: 17–22. 12495858 10.1016/s0166-2236(02)00006-1

[4] Liu P, Qin W, Zhang Y, Tian J, Bai L, et al (2009) Combining spatial and temporal information to explore function-guide action of acupuncture using fMRI. J Magn Reson Imaging 30: 41–46. 19557845 10.1002/jmri.21805

[5] Claunch JD, Chan ST, Nixon EE, Qiu WQ, Sporko T, et al (2012) Commonality and specificity of acupuncture action at three acupoints as evidenced by FMRI. Am J Chin Med 40: 695–712. 22809025 10.1142/S0192415X12500528 PMC3754829

[6] Zhang Y, Liang J, Qin W, Liu P, von Deneen KM, et al (2009) Comparison of visual cortical activations induced by electro-acupuncture at vision and nonvision-related acupoints. Neurosci Lett 458: 6–10. 19442868 10.1016/j.neulet.2009.04.027

[7] Hui KK, Napadow V, Liu J, Li M, Marina O, et al. (2010) Monitoring acupuncture effects on human brain by FMRI. J Vis Exp. 10.3791/1190 PMC3149981 20379133

[8] Huang W, Pach D, Napadow V, Park K, Long X, et al (2012) Characterizing acupuncture stimuli using brain imaging with FMRI–a systematic review and meta-analysis of the literature. PLoS One 7: e32960. 22496739 10.1371/journal.pone.0032960 PMC3322129

[9] Liu J, Qin W, Guo Q, Sun J, Yuan K, et al (2011) Divergent neural processes specific to the acute and sustained phases of verum and SHAM acupuncture. J Magn Reson Imaging 33: 33–40. 21182118 10.1002/jmri.22393

[10] Feng Y, Bai L, Ren Y, Chen S, Wang H, et al (2012) FMRI connectivity analysis of acupuncture effects on the whole brain network in mild cognitive impairment patients. Magn Reson Imaging 30: 672–682. 22459434 10.1016/j.mri.2012.01.003

[11] Liu P, Zhang Y, Zhou G, Yuan K, Qin W, et al (2009) Partial correlation investigation on the default mode network involved in acupuncture: an fMRI study. Neurosci Lett 462: 183–187. 19595739 10.1016/j.neulet.2009.07.015

[12] Hsu SF, Chen CY, Ke MD, Huang CH, Sun YT, et al (2011) Variations of brain activities of acupuncture to TE5 of left hand in normal subjects. Am J Chin Med 39: 673–686. 21721148 10.1142/S0192415X11009111

[13] Qin W, Tian J, Bai L, Pan X, Yang L, et al (2008) FMRI connectivity analysis of acupuncture effects on an amygdala-associated brain network. Mol Pain 4: 55. 19014532 10.1186/1744-8069-4-55 PMC2596101

[14] Hui KK, Marina O, Claunch JD, Nixon EE, Fang J, et al (2009) Acupuncture mobilizes the brain’s default mode and its anti-correlated network in healthy subjects. Brain Res 1287: 84–103. 19559684 10.1016/j.brainres.2009.06.061 PMC3742122

[15] Dhond RP, Yeh C, Park K, Kettner N, Napadow V (2008) Acupuncture modulates resting state connectivity in default and sensorimotor brain networks. Pain 136: 407–418. 18337009 10.1016/j.pain.2008.01.011 PMC2440647

[16] Sporns O, Chialvo DR, Kaiser M, Hilgetag CC (2004) Organization, development and function of complex brain networks. Trends Cogn Sci 8: 418–425. 15350243 10.1016/j.tics.2004.07.008

[17] Grefkes C, Nowak DA, Eickhoff SB, Dafotakis M, Kust J, et al (2008) Cortical connectivity after subcortical stroke assessed with functional magnetic resonance imaging. Ann Neurol 63: 236–246. 17896791 10.1002/ana.21228

[18] Wang L, Yu C, Chen H, Qin W, He Y, et al (2010) Dynamic functional reorganization of the motor execution network after stroke. Brain 133: 1224–1238. 20354002 10.1093/brain/awq043

[19] Askim T, Indredavik B, Vangberg T, Haberg A (2009) Motor network changes associated with successful motor skill relearning after acute ischemic stroke: a longitudinal functional magnetic resonance imaging study. Neurorehabil Neural Repair 23: 295–304. 18984831 10.1177/1545968308322840

[20] Dinomais M, Groeschel S, Staudt M, Krageloh-Mann I, Wilke M (2012) Relationship between functional connectivity and sensory impairment: red flag or red herring? Hum Brain Mapp 33: 628–638. 21391277 10.1002/hbm.21227 PMC6870314

[21] Gao JH, Parsons LM, Bower JM, Xiong J, Li J, et al (1996) Cerebellum implicated in sensory acquisition and discrimination rather than motor control. Science 272: 545–547. 8614803 10.1126/science.272.5261.545

[22] O’Reilly JX, Beckmann CF, Tomassini V, Ramnani N, Johansen-Berg H (2010) Distinct and overlapping functional zones in the cerebellum defined by resting state functional connectivity. Cereb Cortex 20: 953–965. 19684249 10.1093/cercor/bhp157 PMC2837094

[23] Breakspear M, Terry JR, Friston KJ (2003) Modulation of excitatory synaptic coupling facilitates synchronization and complex dynamics in a biophysical model of neuronal dynamics. Network 14: 703–732. 14653499

[24] Grefkes C, Fink GR (2011) Reorganization of cerebral networks after stroke: new insights from neuroimaging with connectivity approaches. Brain 134: 1264–1276. 21414995 10.1093/brain/awr033 PMC3097886

[25] Hummel F, Celnik P, Giraux P, Floel A, Wu WH, et al (2005) Effects of non-invasive cortical stimulation on skilled motor function in chronic stroke. Brain 128: 490–499. 15634731 10.1093/brain/awh369

[26] Murase N, Duque J, Mazzocchio R, Cohen LG (2004) Influence of interhemispheric interactions on motor function in chronic stroke. Ann Neurol 55: 400–409. 14991818 10.1002/ana.10848

[27] He BJ, Snyder AZ, Vincent JL, Epstein A, Shulman GL, et al (2007) Breakdown of functional connectivity in frontoparietal networks underlies behavioral deficits in spatial neglect. Neuron 53: 905–918. 17359924 10.1016/j.neuron.2007.02.013

[28] Nomura EM, Gratton C, Visser RM, Kayser A, Perez F, et al (2010) Double dissociation of two cognitive control networks in patients with focal brain lesions. Proc Natl Acad Sci U S A 107: 12017–12022. 20547857 10.1073/pnas.1002431107 PMC2900657

[29] Fang J, Jin Z, Wang Y, Li K, Kong J, et al (2009) The salient characteristics of the central effects of acupuncture needling: limbic-paralimbic-neocortical network modulation. Hum Brain Mapp 30: 1196–1206. 18571795 10.1002/hbm.20583 PMC6871074

[30] Huang Y, Chen JQ, Lai XS, Tang CZ, Yang JJ, et al. (2013) Lateralisation of cerebral response to active acupuncture in patients with unilateral ischaemic stroke: an fMRI study. Acupunct Med. 10.1136/acupmed-2012-010299 23822904

[31] Schaechter JD, Connell BD, Stason WB, Kaptchuk TJ, Krebs DE, et al (2007) Correlated change in upper limb function and motor cortex activation after verum and sham acupuncture in patients with chronic stroke. J Altern Complement Med 13: 527–532. 17604556 10.1089/acm.2007.6316

[32] Maciocia G (1994) The practice of Chinese medicine: the treatment of diseases with acupuncture and Chinese herbs. Edinburgh, UK: Churchill Livingstone. 342–385 p.

[33] Liu B, Liu X, Chen J, Long Y, Chen ZG, et al (2009) Study on the effects of acupuncture at acupoint and non-acupoint on functional connectivity of different brain regions with functional magnetic resonance imaging. Zhongguo Zhen Jiu 29: 981–985. 20088418

[34] (1999) MONICA Manual.

[35] Cavanna AE, Trimble MR (2006) The precuneus: a review of its functional anatomy and behavioural correlates. Brain 129: 564–583. 16399806 10.1093/brain/awl004

[36] Johansen-Berg H, Dawes H, Guy C, Smith SM, Wade DT, et al (2002) Correlation between motor improvements and altered fMRI activity after rehabilitative therapy. Brain 125: 2731–2742. 12429600 10.1093/brain/awf282

[37] Johansen-Berg H, Rushworth MF, Bogdanovic MD, Kischka U, Wimalaratna S, et al (2002) The role of ipsilateral premotor cortex in hand movement after stroke. Proc Natl Acad Sci U S A 99: 14518–14523. 12376621 10.1073/pnas.222536799 PMC137915

[38] Small SL, Hlustik P, Noll DC, Genovese C, Solodkin A (2002) Cerebellar hemispheric activation ipsilateral to the paretic hand correlates with functional recovery after stroke. Brain 125: 1544–1557. 12077004 10.1093/brain/awf148

[39] Leibing E, Leonhardt U, Koster G, Goerlitz A, Rosenfeldt JA, et al (2002) Acupuncture treatment of chronic low-back pain – a randomized, blinded, placebo-controlled trial with 9-month follow-up. Pain 96: 189–196. 11932074 10.1016/s0304-3959(01)00444-4

[40] Fink MG, Kunsebeck HW, Wippermann B (2000) Effect of needle acupuncture on pain perception and functional impairment of patients with coxarthrosis. Z Rheumatol 59: 191–199. 10929448 10.1007/s003930070080

[41] Molsberger AF, Manickavasagan J, Abholz HH, Maixner WB, Endres HG (2012) Acupuncture points are large fields: the fuzziness of acupuncture point localization by doctors in practice. Eur J Pain 16: 1264–1270. 22492604 10.1002/j.1532-2149.2012.00145.x

[42] Li Y, Liang F, Yang X, Tian X, Yan J, et al (2009) Acupuncture for treating acute attacks of migraine: a randomized controlled trial. Headache 49: 805–816. 19438740 10.1111/j.1526-4610.2009.01424.x

[43] Zhong C, Bai L, Dai R, Xue T, Wang H, et al (2012) Modulatory effects of acupuncture on resting-state networks: a functional MRI study combining independent component analysis and multivariate Granger causality analysis. J Magn Reson Imaging 35: 572–581. 22069078 10.1002/jmri.22887

[44] Moffet HH (2009) Sham acupuncture may be as efficacious as true acupuncture: a systematic review of clinical trials. J Altern Complement Med 15: 213–216. 19250001 10.1089/acm.2008.0356

[45] Wang JJ, Wu ZC (2009) [Thinking about the conclusion of no difference between the acupuncture and sham-acupuncture in the clinically therapeutic effects on migraine abroad]. Zhongguo Zhen Jiu 29: 315–319. 19565742

