# Supplementary material for: Modulatory Effect of Acupuncture at Waiguan (TE5) on the Functional Connectivity of the Central Nervous System of Patients with Ischemic Stroke in the Left Basal Ganglia
Source: PLoS One. 2014 Jun 13;9(6):e96777. doi: 10.1371/journal.pone.0096777 (PMC4057077; doi:10.1371/journal.pone.0096777)
Supplement: Protocol S1 — Trial protocol. (DOC) [file pone.0096777.s002.doc]

Trial protocol

1. Objectives

In this research, cerebral function imaging techniques, i.e. functional magnetic resonance (fMRI) were applied to detect indicators of functional activities in different areas of the brain, to observe brain changes after acupoints and non-acupoint needling or sham needling in pathological conditions, to explore the imaging characteristics of the acupoints needling, to provide some experimental evidence for hypothesis of "specific acupoint-brain relevance", to establish definition model of " specific acupoint-brain relevance ", and to explore essential differences between acupoint and non-acupoint.

1. Background

Acupuncture is one of the most widely used alternative treatment whose curative effect has been approved of by the WHO. However, the mechanism of its effect has not been well characterized to date. Clinical and experimental studies suggest that the modulatory effect might be mediated via central and peripheral nerve system.

Prof. Lai proposed for the first time that stimulating an acupoint might have its specific effects on central nervous system. TE5 is an important traditional acupuncture point common in the treatment of stroke-related motor, neurological and autonomic nerve problems in clinical practice. We hypothesize that acupuncture at TE5 may have specific influence on the functional networks of the CNS comprising sensorimotor areas to help improve the sensorimotor function of the body.

Recently, researches indicate that acupuncture at TE5 could impose some effect on certain brain areas and enhance cooperation between these regions. Therefore, TE5 might closely relate to functional connectivity of the brain which might be a possible mechanism of promoting recovery of stroke patients. Functional connectivity is pattern of statistical dependency resulted from the non-linear dynamics of neurons and neuronal populations within the neuroanatomical substrate which reflexes the existence and the strength of the connectivity between remote brain regions. Modern imaging techniques, fMRI for instance, enable us to analyze the correlation of brain activity signals between distinct brain regions which in turn reflexes functional connectivity.

Generally speaking, we intend to detect the specific influence of acupuncture at TE5 on functional connectivity of central nervous system of patients with ischemic stroke and explore the probable mechanism of acupuncture’s effect on rehabilitation of patients with ischemic stroke.

1. Research design and methods
   1. Subject

Patients with ischemic stroke in left basal ganglia.

- 1. Inclusion criteria

(1) In line with ‘Stroke Diagnosis and Efficacy Assessment Standard’ and diagnostic criteria of ‘Cerebrovascular Disease Diagnosis’ for ischemic encephalopathy;

(2) Significant right hemiplegia and sensory disability;

(3) Stable condition;

(4) Regular diet, not addicted to alcohol, tobacco , coffee and tea, normal sleep and moderate body size;

(5) Receiving usual treatment for the last month and within the experiment process;

(6) Aged between 40 and 65 years old;

(7) Right handedness;

(8) Willing to sign the informed consent.

- 1. Exclusion criteria

(1) Course of disease longer than 1 year;

(2) With acupuncture treatment within1 month prior to the experiment;

(3) With severe heart, liver, or kidney disease, or tumor;

(4) Severe aphasia or psychiatric history of dementia or claustrophobia;

(5) Pregnant women or those in lactation;

(6) With metal in his/her body (such as heart stents); and

(7) With contraindication for acupuncture, such as hemophilia.

- 1. Experiment design

3.4.1 Group allocation

Randomize 24 patients to Waiguan(TE5) group and non-acupoint group, with 12 patients in each group.

3.4.2 Experiment process

(1) choose participants after examination and diagnosis and ask them to sign the informed consent and case history registry form one week before the experiment begins;

(2) make an arrangement with Imaging Center of Nanfang Hospital 2 days before the experiment, then inform the participants the experiment time and ask whether their condition deteriorates;

(3) on the morning of experiment day, participants will be picked up and carried to Imaging Center of Nanfang Hospital and register for scanning;

(4) ensure that participants haven’t got metal stuffs or magcard with them and have them rest for 10 min or so. Tell the participants to remain calm and must not shake their head while scanning. Mark the stimulating spot on their forearm;

(5) acupuncture and scanning program:

1)The fMRI brain scan is conducted on a 3.0-T whole-body scanner (GE Signa) with a standard head coil.

2)Participants are prevented from experiencing auditory and visual activities via earplugs and eyeshades, respectively.

3)The scanning procedure begins after participants resting on bed for 5min. Firstly, a 2min42s scanning procedure is done to collect 3D anatomical images.

4)Then participants in each group are given sham acupuncture stimulus and verum acupuncture in turn, being scanned at the same time, each stimulus lasting 6min30s. Between the two stimuli, there is a 6min2s’ interval.

3.4.3 Acupuncture

(1) Verum acupuncture stimulation

When stimulating, a sterile silver needle is inserted vertically into the skin for a depth of 15±2mm. After needling sensation (de qi) is acquired, assured by the acupuncturist feeling of increased resistance to further inserting, the needle is twisted for ±180° evenly at a frequency of 60 circles per min.

(2) Sham acupuncture stimulation

The procedure is to push the end of the needle with its tip out of the tube within 1mm and touching the skin.

3.4.4 Data analysis

Preprocessing steps are implemented in AFNI (Cox, 1996; http://afni.nimh.nih.gov/afni). The functional images from each run are aligned, slice timing corrected, temporally standardized, space smoothed (6mm full width at half maximum Gaussial kernal) and transformed into Talaraich space (Talairach and Tournoux, 1988).

6 regions of interest(ROI) from bilateral hemisphere are chosen including areas related to motor ability(Brodmann area(BA)4 and BA6) sensations(BA1, BA2, BA3, BA5 and BA7) and basal ganglia area. The low frequency BOLD correlations (0.01~0.1 Hz), i.e. functional connectivity, between the time series of a given seed and of all voxels in the brain are partial correlated by Pearson correlation. Those voxels that have significant correlation with ROIs are extracted, with multiple-comparison error corrected by Monte Carlo simulation. Correlation coefficient maps thresholded at P < 0.05 for both the acupuncture and tactile conditions are generated and transformed to the Fisher’ Z for group analysis.

Patients with ischemic stroke

A week before experiment

Those matching the including criteria sign the informed consent and case history registry after examination and diagnosis

Waiguan group(n=12)：

rest 5min—→scan for 2min42s—→sham acupuncture+fcMRI scanning for 6min30s—→3D scanning for 6min2s—→verum acupuncture+ fcMRI scanning for 6min30s

Non-acupoint group(n=12)：

rest 5min—→scan for 2min42s—→sham acupuncture+fcMRI scanning for 6min30s—→3D scanning for 6min2s—→verum acupuncture+fcMRI scanning for 6min30s

Image processing and functional connectivity analysis

Conclusion for effects of acupuncture at Waiguan(TE5)
